# Editorial: Application of multimodal imaging combined with artificial intelligence in eye diseases

**DOI:** 10.3389/fnins.2023.1287762

**Published:** 2023-09-19

**Authors:** Zhi Wen, Yuchen Chen, Vijaya Prakash Krishnan Muthaiah, Xin Huang

**Affiliations:** ^1^Department of Radiology, Renmin Hospital of Wuhan University, Wuhan, Hubei, China; ^2^Department of Radiology, Nanjing First Hospital, Nanjing Medical University, Nanjing, Jiangsu, China; ^3^Department of Rehabilitation Sciences, School of Public Health and Health Professions, University at Buffalo, Buffalo, NY, United States; ^4^Department of Ophthalmology, Jiangxi Provincial People's Hospital, The First Affiliated Hospital of Nanchang Medical College, Nanchang, Jiangxi, China

**Keywords:** eye disease, optic atrophy, visual pathway, OCT, OCTA, AI, fMRI

## Introduction

Vision is the most crucial sensory system for human beings to process external information. The retina, optic nerve, and visual pathway (lateral geniculate body, optic radiation, and visual cortex) are all necessary for the complete transmission of visual information. Numerous eye conditions, including glaucoma, diabetic retinopathy, optic neuritis, and hereditary optic neuropathy result in vision loss, and as a result, they remain a key field of study for both clinical and pre-clinical research. Multimodal imaging techniques, such as optical coherence tomography (OCT), optical coherence tomography angiography (OCTA), and functional magnetic resonance imaging (fMRI), give essential biological indicators in the diagnosis of ophthalmic diseases. In fact, combining imaging with artificial intelligence (AI) may enhance diagnostic precision and accuracy, detect imaging biomarkers, develop cutting-edge tools that will impact clinical practice and benefit patient outcomes.

This Research Topic on “*Application of multimodal imaging combined with artificial intelligence in eye diseases*” included 19 articles. It covered a range of AI applications in fundus images, OCT, anterior segment images, infrared videos from eye movement recorders, and steady-state visual evoked potentials (SSVEPs) in order to classify eye diseases, or to find neuroimaging indicators. Other papers reviewed the literature, identified indicators from animal model studies, and determined the prognostic factors of transnasal endoscopic optic decompression. In this editorial, we give an overview of these studies and group them according to the study design.

## AI-aided diagnosis

Retinal atrophy is a crucial assessment indicator as it is correlated with the severity of myopia. For 2D fundus images, Chen et al. developed an attention-aware retinal atrophy segmentation network based on UNet structure called ARA-Net. To deal with blurred boundaries and irregular shapes of the retinal atrophic region, they proposed a novel skip self-attention (SSA) connection block. They also proposed a multi-scale feature flow (MSFF) between the SSA connection blocks, greatly enhancing the self-attention mechanism's capacity. The Pathological Myopia (PALM) dataset has been used to validate the proposed method, which performs noticeably better than other methods. A new deep learning model called MyopiaDETR was put up by Li M. et al. for the diagnosis of pathological myopia (PM) using 2D fundus image data. The architecture of Detection Transformer (DETR) allows it to effectively tackle the issue of morphology irregularity. An attentional feature pyramid network (FPN) increases the difference in feature intensity the between foreground and background. The experimental results show that their model outperforms other state-of-the-art object detectors in terms of localization and classification performance on the iChallenge-PM dataset.

Huang et al. introduces GABNet, a novel lightweight classification network model based on the global attention block (GAB) for OCT. By using their method, classification accuracy is increased over the EfficientNetV2B3 network model by 3.7%. Gan, Wu, et al. proposed an AI method based on multifeature fusion to enable automatic macular edema (ME) classification on spectral domain OCT (SD-OCT) images. With an accuracy of 93.8%, the support vector machine (SVM) model performed the best when compared to other classification models.

Gan, Liu, et al. and Li, Huang, Peng developed AI segmentation platforms with a deep transfer-learning algorithm and multi-feature fusion by using anterior segment images for automatic cortical cataract staging and fungal keratitis diagnosis, respectively. One is based on a method of automatic segmentation, whereas the other is based on a method of manual segmentation. While the automatic segmentation platform can stage cataracts and diagnose fungal keratitis more quickly, the manual segmentation platform can do so more accurately. In addition, Gan, Chen, et al. developed an AI model based on ensemble DL that was combined with four benchmark models (the Resnet18, Alexnet, Googlenet, and Vgg11) for identifying pterygium that need to be surgically removed. The ensemble DL model exceed all other models in terms of accuracy and area under the curve (AUC).

Li and Yang demonstrated that torsional nystagmus can be recognized by deep learning networks models. They used convolution neural network to extract the frame features of the infrared video sequence from eye movement recorders, and classified the obtained vector sequence.

For SSVEPs, Wan, Li, et al. proposed a transformer–based EEGformer analysis model to capture the electroencephalogram (EEG) characteristics in a unified manner. Across three EEG datasets [BETA, SJTU emotion EEG dataset (SEED), depressive EEG database (DepEEG)] the EEGformer achieves the best classification performance. This finding suggests that the rationality of model architecture and learning EEG characteristics in a unified manner can improve model classification performance. Another study by Wan, Cheng, et al. propose a deep neural network called GDNet-EEG for SSVEP stimulation frequency recognition that uses group depth-wise convolutional filtering to extract regional characteristics from raw EEG data. The findings show that GDNet-EEG surpasses the existing deep learning models to process EEG data on two publicly available SSVEPs datasets (largescale benchmark and BETA dataset) and their merged dataset.

For resting-state fMRI, Ji, Wang, et al. used the amplitude of low-frequency fluctuation (ALFF) in conjunction with sliding window approach to assess the changes of dynamic neural activity in patients with retinal detachment. Based on dALFF values, the overall accuracies of SVM classification were good under three different time windows. In patients with primary angle-closure glaucoma, Li, Huang, Peng, Liang, et al. identified changes in functional connectivity (FC) with primary visual cortex (V1), and found the increased FC between V1 and calcarine. However, the discrimination of PACG from healthy controls (HC) was poor when utilizing the SVM method and the dFC map as the classification feature.

## Neuroimaging indicators

Ji, Huang, et al. explore differences in static FC (sFC) and dynamic FC (dFC) alteration patterns in the V1 among patients with high myopia and HCs via seed-based FC analysis. This disturbance suggests that patients with high myopia could exhibit impaired cognitive and emotional processing functions, top-down control of visual attention, and visual information processing functions. Pang et al. compared retinal OCT and optic nerve diffusion tensor imaging (DTI) parameters in patients with non-functioning pituitary adenoma (NFPA). The degree of adverse changes in OCT and DTI parameters was found to be stronger in the severe compression group than that in the mild compression group. Moreover, the fractional anisotropy (FA) value of the optic chiasma has a high diagnostic ability for visual pathway impairment. A literature review by Wang et al. described the use of DTI technology in glaucoma in humans and animal models, with the advancement of DTI technology and its coupling with artificial intelligence, DTI represents a potential future for MRI technology in glaucoma research.

## Indicators from animal models

Accurate axial length (AL) measurement is crucial for developing animal models of myopia. The accuracy of Quantel A-B scan, OD-1 A scan, and vernier caliper were compared by Wu et al. for measuring AL in Sprague Dawley rats. They found that Quantel A-B scan might be more accurate than OD-1 A scan. AL and refractive error (RE) are both influenced by gender. Duan et al. employed OCT to assess the changes in retina thickness after shape deprivation in myopic mice, and found considerably thinner retina, nerve fiber layer, inner nuclear layer, and outer nuclear layer. In addition, Shi et al. found epidermal growth factor-containing fibulin-like extracellular matrix protein 1 (EFEMP1) may have a role in the choroidal thickness regulation by using guinea pig model for myopia.

Last but not least, Tu et al. investigate the clinical effectiveness and prognostic variables of trans nasal endoscopic optic decompression in the treatment of traumatic optic neuropathy. The prognosis is highly dependent on the presence of residual light perception and the timing of surgery within 7 days.

As we draw to a close, we wish to emphasize the need of making more efforts to enhance the technical, clinical, and preclinical advances described in this Research Topic. In future, with the aid of AI, it will be feasible to provide a comprehensive description of eye diseases by integrating data from multidimensional modalities, as well as those from patients and animal models. Furthermore, it is hoped that the existing large-scale image dataset will be useful in this regard.

## Author contributions

ZW: Writing—original draft. YC: Writing—review and editing. VK: Writing—review and editing. XH: Writing—review and editing.

